# Impact of sarcopenia on variceal rebleeding in patients after endoscopic therapy: a multicenter retrospective cohort study based on propensity score matching

**DOI:** 10.1080/07853890.2024.2349180

**Published:** 2024-05-03

**Authors:** Yongshuai Liu, Huijun Chang, Yunqing Zeng, Yuanyuan Liu, Jinhou Li, Yong Chen, Yanjing Gao

**Affiliations:** a Department of Gastroenterology, Qilu Hospital of Shandong University, Jinan, Shandong, China; b Department of Gastroenterology, Taian City Central Hospital, Taian, Shandong, China; c Department of Gastroenterology, Shandong Provincial Hospital Affiliated to Shandong First Medical University, Jinan, Shandong, China

**Keywords:** Liver cirrhosis, endoscopic therapy, rebleeding, sarcopenia

## Abstract

**Background:**

Sarcopenia is a common complication of liver cirrhosis and can be used for predicting dismal prognostic outcomes. This study aimed to evaluate the role of sarcopenia in rebleeding and mortality of liver cirrhosis patients after endoscopic therapy.

**Methods:**

The liver cirrhosis patients who received endoscopic treatment were enrolled. Propensity score matching (PSM) was used to overcome selection bias. Two-year rebleeding episodes and mortality after endoscopic therapy were recorded.

**Results:**

A total of 109 (32.4%) sarcopenia patients were reported. Before PSM, the frequency of rebleeding was significantly higher in the sarcopenia group relative to the non-sarcopenia group (41.3% vs. 15.9%, *p* < 0.001). Moreover, the multivariable analysis revealed that sarcopenia (*p* < 0.001, HR:2.596, 95% CI 1.591–4.237) was independently associated with a 2-year rebleeding episode. After PSM, the sarcopenia group exhibited an increased rebleeding rate as compared with non-sarcopenia group (44.4% vs. 15.3%, *p* < 0.001). According to multivariable analysis, sarcopenia (*p* < 0.001, HR:3.490, 95% CI 1.756–6.938) was identified as a significant predictor for 2-year rebleeding.

**Conclusion:**

Sarcopenia was significantly associated with a high 2-year rebleeding rate in liver cirrhosis patients after endoscopic treatment. Therefore, the precise evaluation of a patient’s nutritional status, including sarcopenia becomes mandatory before endoscopic treatment.

## 1. Introduction

Esophagogastric variceal bleeding (EGVB), a common portal hypertension-related complication, is a major factor causing mortality in cirrhotic patients [1]. The surviving patients and those recovering from a first acute variceal bleeding episode also display increased rebleeding and mortality risks (approximately 60% and 33% in the first year) without secondary prophylaxis [2]. Combining nonselective β-blockers (NSBBs) with endoscopic treatment can effectively prevent rebleeding and prolong the survival time [3]. Several studies reported that EGVB risk following endoscopic treatment is about 7.8–29%; and fatal variceal rebleeding might occur after endoscopic treatment [4,5]. Therefore, elucidating the risk factors for identifying high-risk patients undergoing endoscopic treatment is of great importance.

In clinical practice, hepatic venous pressure gradient (HVPG), Child-Pugh and the model for end-stage liver disease (MELD) scores have been widely demonstrated as strong prognostic indicators for recurrent variceal bleeding and survival in patients diagnosed with cirrhosis [3,6]. Nevertheless, the major limitation of these indicators is the lack of nutritional evaluation and functional status of patients. Sarcopenia is a progressive loss of muscle mass, strength, and functional capacity reflecting protein-energy malnutrition and is recognized as a quantitative and objective method of assessing a patient’s nutritional status [7,8]. There has been growing attention towards the significant impact of sarcopenia, which may be associated with elevated portal pressure, resulting in the development of hepatic decompensation and ultimately leading to death [9,10]. For instance, Zeng et al. found that sarcopenia patients displayed poor liver function and higher complication rates, such as susceptibility to infections and upper gastrointestinal varices [11]. As discovered by Nardelli et al. sarcopenia showed an independent association with minimal hepatic encephalopathy (HE) and overt HE risk among cirrhotic patients [12]. A recent study found that sarcopenia was a predictive factor of decompensation with ascites, independently from the presence of clinically significant portal hypertension [13].

Considering sarcopenia could be related to the prognosis of patients with liver cirrhosis, it is hypothesized that sarcopenia may be able to predict the clinical outcome of cirrhosis patients after endoscopic therapy. Until date, few studies directly investigated the association between sarcopenia and prognosis in cirrhotic patients with variceal bleeding undergoing endoscopic therapy. The objective of this study aimed to evaluate the role of CT-assessed sarcopenia, in variceal bleeding patients treated by endoscopic therapy for identifying high-risk patients.

## 2. Materials and methods

### 2.1. Study population

We retrospectively evaluated liver cirrhosis patients receiving secondary prevention of variceal rebleeding at three hospitals between January 2016 to May 2021. The exclusion criteria were: (1) patients age <18 or >80 years; (2) those who previously received endoscopic treatment, liver transplantation, or transjugular intrahepatic portosystemic shunt (TIPS) placement; (3) patients with hepatocellular carcinoma and additional extrahepatic cancer; (4) patients not receiving abdominal CT before the treatment; (5) those with a follow-up period of <2 years. Our study protocol was carried out in line with the Declaration of Helsinki statements and was approved by the research ethics committee of Qilu Hospital of Shandong University (NO. KYLL-202310-029). Informed consent was not required due to the study’s retrospective nature.

### 2.2. Clinical and laboratory data

The patient’s baseline features were extracted at the time of the first endoscopic treatment like age, gender, body mass index (BMI), the primary cause of cirrhosis, and coexisting illnesses. Routine laboratory values like hemoglobin (Hb), platelet count, total bilirubin, serum albumin, prothrombin time (PT) and international normalized ratio (INR) were obtained upon admission. Moreover, ascites was categorized into present or absent conditions. Additionally, we recorded Child-Pugh and the model for end-stage liver disease (MELD) scores. Two physicians were responsible for independent data collection, which was verified by a third researcher.

### 2.3. Endoscopic and NSBBs treatment

Endoscopic dense ligation was completed with commercially available multiband devices after intravenous anesthesia. Additionally, varix ligation was carried out from the cardia to the oral side. The band number was decided according to varices with red plugs and bleeding signs measured by the operator. Gastroesophageal varicose patients were given endoscopic tissue adhesives like N-butyl cyanoacrylate when necessary. All endoscopic characteristics were evaluated following endoscopic recording rules for esophagogastric varices. Endoscopic variceal ligation (EVL) was conducted at 28–42 days intervals till eradication [14]. Moreover, NSBBs (carvedilol or propranolol) at the standard doses were used in patients following Baveno VI recommendations in the absence of any contraindication, with systolic blood pressure and heart rate being kept at >90 mmHg and >55 beats/min, respectively.

### 2.4. Skeletal muscle measurement and sarcopenia diagnosis

Two experienced reviewers obtained patients’ abdominal CT images and determined the cross-sectional skeletal muscle areas at the third lumbar vertebra (L3) level as −29 to +150 Hounsfield units. SliceOmatic software (version 5.0, TomoVision, Magog, Quebec, Canada) was used for image analysis. The cross-sectional skeletal muscle areas were normalized to stature (cm^2^/m^2^) according to the previous description, which represented the L3 skeletal muscle index (L3 SMI). The definition of sarcopenia was made in line with the threshold verified in the previous study for cirrhotic patients: L3 SMI: <32.5 cm^2^/m^2^ and <44.77 cm^2^/m^2^ for females and males, respectively [11].

### 2.5. Study endpoints and follow-up

Rebleeding following an initial endoscopic treatment was our study’s primary outcome, while death event was the secondary endpoint. The following conditions in follow-up were deemed as rebleeding episodes: (1) presence of clinically-significant bleeding was deemed as relapsed melena or hematemesis, decreased Hb content by ≥3 g/L, and (2) active gastric or esophageal variceal rebleeding discovered in endoscopic follow-up. All patients were regularly followed up for 2 years util death, liver transplantation or end of follow-up. Additionally, the rebleeding rate and time of death following initial endoscopic treatment were measured.

### 2.6. Statistical analysis

SPSS 26.0 software (SPSS, Chicago, IL, US) was employed for data analysis. Quantitative results were reported by means ± standard deviation (SD) or median and interquartile range (IQR) and compared by Mann-Whitney U-test or Student’s t-tests. The categorical data were reported by counts and percentages and compared by Chi-square test. Propensity scoring matching (PSM) was used for the control of selection bias and was performed using binary logistic regression to generate a propensity score for each patient who had or did not have sarcopenia. Using the nearest-neighbor method without replacement for propensity score matching, pairs of patients were matched with a match tolerance of 0.02 and a ratio of 1:1. Variables included in the propensity model were age, sex, BMI, hemoglobin, total bilirubin, serum albumin, Child-Pugh score, ascites. We selected these variables because they resulted in significant differences prior to PSM. Kaplan-Meier curves with log-rank tests were applied in estimating the impact of sarcopenia on rebleeding after endoscopic treatment. All factors independently predicting 2-year rebleeding were identified by univariable/multivariable Cox regression analysis. Possible risk factors, combined with factors satisfying *p* < 0.1 from univariable Cox regression analysis were incorporated into multivariable regression analysis for evaluating hazard ratios (HRs). The *p* < 0.05 (two-sided) values were statistically significant.

## 3. Results

**Figure 1.**
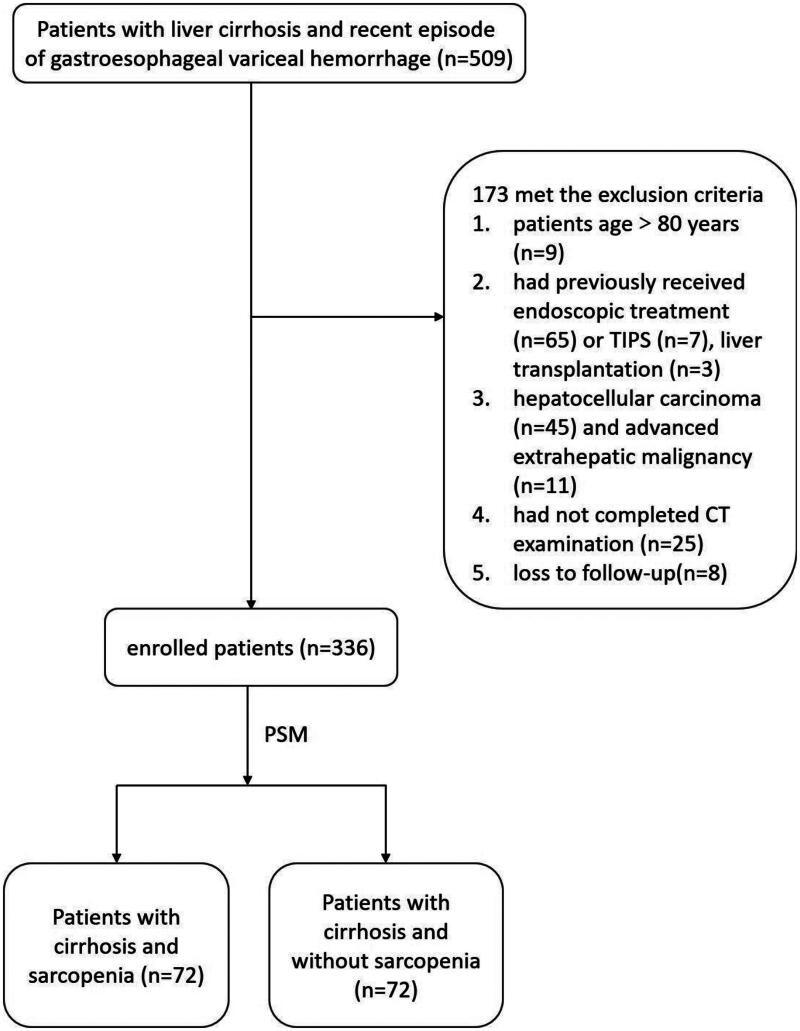
Flowchart. Retrospective selection process of patients.

From January 2016 to May 2021, 509 liver cirrhosis patients underwent endoscopic therapy to prevent gastroesophageal variceal rebleeding; a total of 173 patients were excluded. Finally, we enrolled 336 patients for eventual analysis. Figure 1 displays the flow chart of study screening.

### 3.1. Patients’ features

Table 1 displays the basic features of 336 patients. Of them, 109(32.4%) patients had sarcopenia. The mean age of all patients was 55 years, with male predominance (*n* = 208, 61.9%). Hepatitis B was the primary etiological factor for liver cirrhosis, and 194 cases (57.7%) had a Child-Pugh grade of A. Endoscopic therapy plus NSBBs was carried out in 248 (73.8%) patients.

**Table 1.** Clinicopathologic characteristics of 336 patients with liver cirrhosis.

Variable	All patients (*n* = 336)
Age (years)	55 ± 10.49
Male, n (%)	208(61.9%)
BMI (kg/m^2^)	23.39 ± 3.24
Etiology, n (%)	
Hepatitis B	168(50%)
Hepatitis C	15(4.5%)
Alcohol	33(9.8%)
Autoimmune	31(9.2%)
Others	85(26.5%)
Laboratory finding	
Hemoglobin (g/L)	90.19 ± 21.47
Platelet count (10^9^/L)	104.62 ± 80.22
Total bilirubin (μmol/L)	18.31 ± 10.83
ALT (U/L)	27.61 ± 26.54
AST (U/L)	32.71 ± 20.18
Serum albumin (g/L)	36.25 ± 4.63
PT (s)	14.47 ± 2.05
INR	1.28 ± 0.19
Child-Pugh score	6.41 ± 1.44
Child-Pugh A, n (%)	194(57.7%)
MELD score	9.82 ± 2.29
Endoscopic features (F1/F2/F3)	0/23/313
Prevalence of PVT, n (%)	132(39.3%)
Ascites, n (%)	150(44.6%)
Number of endoscopic treatments	2.15 ± 0.884
Number of bands	9.4 ± 3.165
NSBBs used, n (%)	248(73.8%)
sarcopenia, n (%)	109(32.4%)

Data are means ± standard deviations.

BMI, body mass index; ALT, alanine aminotransferase; AST, aspartate aminotransferase; PT, prothrombin time; INR, international normalised ratio; MELD, Model For End Stage Liver Disease; PVT, portal vein thrombosis; NSBBs, nonselective beta-receptor blockers.

### 3.2. Comparison of features between sarcopenia and non-sarcopenia groups

Patients were separated into a sarcopenia and a non-sarcopenia group (Table 2). The sarcopenia group consisted of 109 patients, whereas the non-sarcopenia group consisted of 227 patients. Few sarcopenia group variables, such as BMI, serum albumin, and Hb were reduced relative to the non-sarcopenia group (*p* < 0.05). However, male predominance, ascites, total bilirubin count, Child-Pugh score, and ages in the sarcopenia group relatively increased when compared to the non-sarcopenia group (*p* < 0.05). The etiology of liver cirrhosis, PVT incidence, platelet count, PT, INR, serum creatinine, and NSBBs used for the patients with and without sarcopenia were not significantly different. Regarding the endoscopic features, there was no significant difference in the endoscopic features, treatment numbers and number of bands.

**Table 2.** Clinical and endoscopic features of cirrhotic patients with and without sarcopenia before and after propensity score matching.

Variables	Before PSM			After PSM		
	Sarcopenia (*n* = 109)	Without sarcopenia (*n* = 227)	*p* value	Sarcopenia (*n* = 72)	Without sarcopenia (*n* = 72)	*p* value
Age (years)	56.77 ± 10.42	54.16 ± 10.44	0.033	54.28 ± 10.22	54.56 ± 10.64	0.873
Male, n (%)	76(69.7%)	132(58.1%)	0.041	47(65.3%)	43(59.7%)	0.491
BMI (Kg/m^2^)	21.78 ± 2.77	24.17 ± 3.16	<0.001	22.53 ± 2.74	22.14 ± 2.77	0.4
Etiology, (n%)			0.151			0.212
Hepatitis B	49(45%)	119(52.4%)		33(45.8%)	37(51.4%)	
Hepatitis C	6(5.5%)	9(4%)		2(2.8%)	5(6.9%)	
Alcoholic	12(11%)	21(9.3%)		7(9.7%)	11(15.3%)	
Autoimmune	16(14.7%)	15(6.6%)		10(13.9%)	4(5.6%)	
Others	26(23.9%)	63(27.8%)		20(27.8%)	15(20.8%)	
Hemoglobin (g/L)	86.86 ± 19.86	91.78 ± 22.07	0.049	86.82 ± 20.17	86.90 ± 18.89	0.980
Platelet count (10^9^/L)	95.22 ± 69.75	109.14 ± 84.56	0.112	102.44 ± 80.12	105.36 ± 79.04	0.826
ALT (U/L)	25.6 ± 22.25	28.57 ± 28.36	0.337	27.32 ± 24.51	25.38 ± 20.29	0.605
AST (U/L)	33.23 ± 23.62	32.45 ± 18.36	0.763	32.9 ± 20.89	32.31 ± 18.26	0.855
Total bilirubin (μmol/L)	19.73 ± 11.92	17.63 ± 10.23	0.096	18.97 ± 10.55	18.51 ± 11.27	0.803
Serum albumin (g/L)	35.24 ± 4.62	36.73 ± 4.57	0.005	35.57 ± 4.93	35.93 ± 4.83	0.660
PT(s)	14.71 ± 2.08	14.35 ± 2.04	0.133	14.61 ± 1.99	14.75 ± 2.03	0.668
INR	1.3 ± 0.18	1.27 ± 0.19	0.166	1.29 ± 0.17	1.31 ± 0.19	0.515
Child-Pugh class, n (%)			<0.001			0.898
A	48(44%)	146(64.3%)		36(50%)	36(50%)	
B	57(52.3%)	75(33%)		34(47.2%)	33(45.8%)	
C	4(3.7%)	6(2.6%)		2(2.8%)	3(4.2%)	
Child-Pugh score	6.81 ± 1.42	6.22 ± 1.41	<0.001	6.67 ± 1.42	6.61 ± 1.56	0.824
MELD score	10.1 ± 2.15	9.69 ± 2.35	0.124	9.96 ± 2.04	10.06 ± 2.34	0.789
Endoscopic features, n (%)			0.831			0.479
F2	10(9.2%)	14(6.2%)		9(12.5%)	12(16.7%)	
F3	99(90.8%)	213(93.8%)		63(87.5%)	60(83.3%)	
Number of endoscopic treatments	2.12 ± 0.87	2.17 ± 0.89	0.611	2.15 ± 0.93	1.99 ± 0.88	0.271
Number of bands	9.07 ± 3.34	9.56 ± 3.07	0.202	9.32 ± 3.41	9.65 ± 2.95	0.531
Ascites, n (%)	59(54.1%)	91(40.1%)	0.015	36(50%)	36(50%)	1
PVT, n (%)	48(44%)	84(37%)	0.217	26(36.1%)	32(44.4%)	0.308
NSBBs used, n (%)	84(77.1%)	164(72.2%)	0.347	57(79.2%)	54(75%)	0.552

Data are means ± standard deviations.

BMI, body mass index; ALT, alanine aminotransferase; AST, aspartate aminotransferase; PT, prothrombin time; INR, international normalised ratio; MELD, Model For End Stage Liver Disease; PVT, portal vein thrombosis; NSBBs, nonselective beta-receptor blockers.

PSM was carried out to reduce the selection bias, which yielded 72 matched pairs. Clinicopathological features were not significantly different between the two groups (*p* > 0.05). Table 2 displays the patients’ features post-PSM.

### 3.3. Rebleeding and mortality after endoscopic treatment

**Figure 2.**
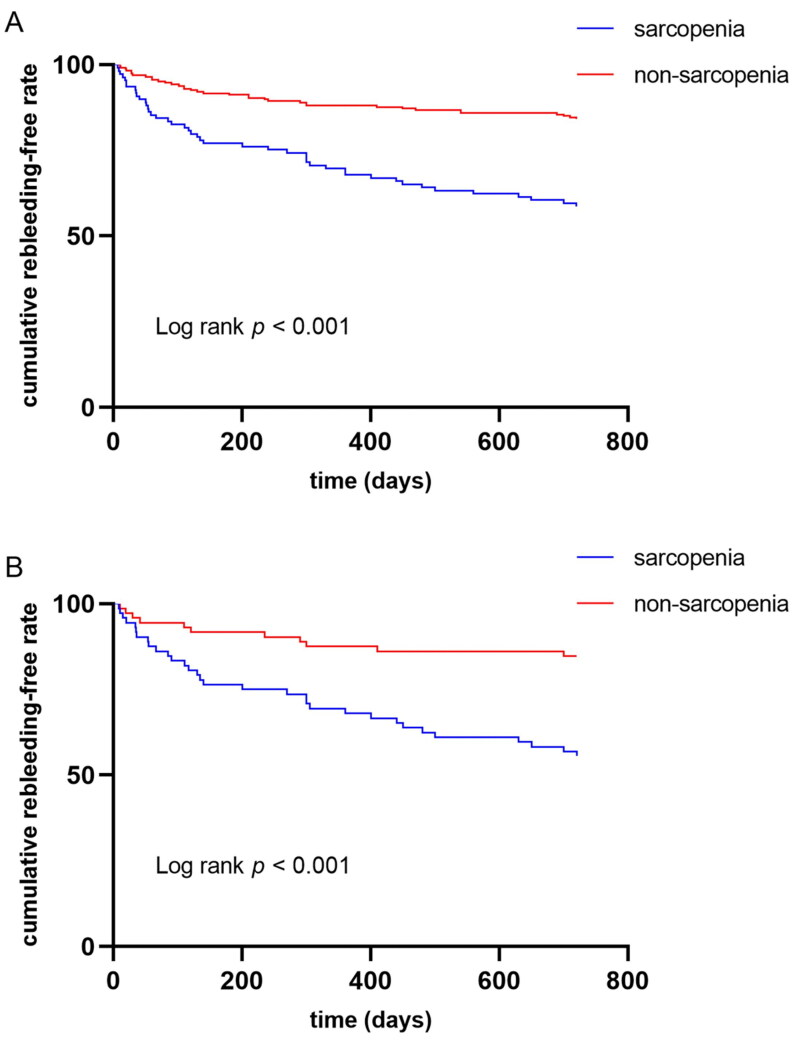
(A) Kaplan–Meier curves of variceal rebleeding 2-year post-operative rebleeding before matching; (B) Kaplan–Meier curves of variceal rebleeding 2-year post-operative rebleeding after matching.

During the 2-year follow-up after the first endoscopic treatment, recurrent hemorrhage occurred in 81 (24.1%) patients. The sarcopenia group showed an increased 2-year rebleeding rate compared with the non-sarcopenia group (2-year rebleeding 41.3% vs. 15.9%, *p* < 0.001). However, rebleeding rates were compared by the Kaplan-Meier curve and log-rank test at diverse times in both groups. The cumulative rebleeding risk of the sarcopenia group significantly increased at 2 years (*p* < 0.001 upon log-rank test, Figure 2A). After rebleeding episodes, 67 patients underwent endoscopic treatment, 8 patients received conservative therapy, 2 patients took TIPS, and 4 died because of uncontrolled bleeding.

Simultaneously, 11 (3.0%) patients died, including 4 and 6 patients who died due to uncontrolled bleeding and dismal liver function. However, one patient died of spontaneous bacterial peritonitis (SBP). The rate of 2-year mortality (5.5% vs. 2.2%, *p =* 0.206) was comparable in both groups.

After PSM, the sarcopenia group showed an increased 2-year rebleeding rate as compared with the non-sarcopenia group (2-year rebleeding 44.4% vs. 15.3%, *p* < 0.001). Moreover, the sarcopenia group’s cumulative rebleeding risk significantly elevated at 2 years (*p* < 0.001 upon log-rank test, Figure 2B). Furthermore, the 2-year mortality rate (5.6% vs. 4.2%, *p =* 0.698) was comparable in both groups.

### 3.4. Risk factors for 2-year rebleeding between the sarcopenia and non-sarcopenia group

Univariable Cox analysis variables significantly associated with 2-year rebleeding are displayed in Table 3. The albumin (*p =* 0.003), Hb (*p =* 0.003), PT (*p =* 0.006), INR (*p =* 0.007), Child-Pugh score (*p* < 0.001), MELD score (*p =* 0.008), ascites (*p =* 0.005), sarcopenia (*p* < 0.001) and NSBBs (*p =* 0.018) were the potential factors that predicted 2-year rebleeding in univariable regression analysis. As revealed by multivariable regression incorporating univariable regression variables of *p* < 0.1, Child-Pugh score (*p =* 0.032, HR: 1.175, 95% CI 1.014–1.361) and sarcopenia (*p* < 0.001, HR:2.596, 95% CI 1.591–4.237) were significant risk factors associated with a 2-year rebleeding episode. Thus, Hb (*p =* 0.028, HR: 0.987, 95% CI 0.976–0.999) and NSBBs (*p =* 0.019, HR: 0.578, 95% CI 0.366–0.913) were considered as the protective elements related with a decreased rebleeding rate.

**Table 3.** Competing risk factor of 2-year rebleeding in all patients.

Variables	Univariate analysis HR (95%CI)	*p* value	Multivariate analysis HR (95%CI)	*p* value
Albumin	0.928(0.883–0.975)	0.003		
ALT	1.007(1.0–1.014)	0.055		
Total bilirubin	1.016(0.998–1.034)	0.08		
Hemoglobin	0.983(0.972–0.994)	0.003	0.987(0.976–0.999)	0.028
PT	1.155(1.041–1.281)	0.006		
INR	4.9(1.548–15.512)	0.007		
Child-Pugh score	1.299(1.139–1.482)	<0.001	1.175(1.014–1.361)	0.032
MELD score	1.13(1.033–1.237)	0.008		
Ascites	1.893(1.217–2.943)	0.005		
sarcopenia	3.134(1.886–5.209)	<0.001	2.754(1.759–4.313)	<0.001
PVT	1.079(0.693–1.681)	0.737		
NSBBs used	0.579(0.367–0.912)	0.018	0.578(0.366–0.913)	0.019

CI, confidence interval; HR, hazard ratio; ALT, alanine aminotransferase; PT, prothrombin time; INR, international normalised ratio; MELD, Model For End Stage Liver Disease; PVT, portal vein thrombosis; NSBBs, nonselective beta-receptor blockers.

Table 4 displays Cox regression analyses of rebleeding among 144 matched patients. As revealed by univariable regression, sarcopenia (*p* < 0.001), albumin (*p =* 0.048), Child-Pugh score (*p =* 0.023), and NSBBs (*p =* 0.029) were considered potential 2-year rebleeding predictors. Based on multivariable regression analysis, sarcopenia (*p* < 0.001, HR:3.490, 95% CI 1.756–6.938) and Child-Pugh scores (*p =* 0.017, HR: 1.254, 95% CI 1.042–1.510) were independent predictors of 2-year rebleeding episodes.

**Table 4.** Competing risk factor of 2-year rebleeding in matched patients.

Variables	Univariate analysis HR (95%CI)	P value	Multivariate analysis HR (95%CI)	P value
Albumin	0.936(0.877–0.999)	0.048		
Total bilirubin	1.008(0.981–1.037)	0.559		
Hemoglobin	0.986(0.971–1.002)	0.082		
Child-Pugh score	1.234(1.029–1.480)	0.023	1.254(1.042–1.510)	0.017
MELD score	1.046(0.911–1.20)	0.524		
Ascites	1.749(0.949–3.225)	0.073		
NSBBs used	0.520(0.275–0.985)	0.045		
sarcopenia	3.134(1.886–5.209)	<0.001	3.490(1.756–6.938)	<0.001

CI, confidence interval; HR, hazard ratio; MELD, Model For End Stage Liver Disease; NSBBs, nonselective beta-receptor blockers.

## 4. Discussion

Sarcopenia is a common complication of liver cirrhosis and appear to be correlated strongly with the severity of cirrhosis [15]. In one study, 22.5% of Chinese liver cirrhosis patients developed sarcopenia [11], which is lower than our findings (32.4%). The reason for this may be that all cirrhosis patients included in our study were complicated with EGVB. In the present study, we thoroughly examined how sarcopenia affected rebleeding and mortality of liver cirrhosis patients following endoscopic therapy. Our results revealed that the rebleeding rate after endoscopic therapy significantly increased in the sarcopenia group (45/109, 41.3%) relative to the non-sarcopenia group (36/227, 15.9%). Based on univariable/multivariable regression analysis and PSM, sarcopenia was a significant predictive marker for rebleeding. Thus, we suggested that a proper evaluation of the presence or absence of sarcopenia might identify a high variceal rebleeding risk in patients undergoing endoscopic therapy.

The relationship of sarcopenia with variceal bleeding among liver cirrhosis patients has rarely been reported and showed contrasting findings on this topic. On the one hand, the presence of esophageal varices show a strong correlation with sarcopenia [16]. Lattanzi et al. observed that sarcopenia was a major predictive factor for refractory variceal bleeding and an independent factor in predicting portal hypertension severity like variceal size and using NSBB drugs among noncirrhotic portal hypertension patients [10]. On the other hand, other studies observed that prognostic role of sarcopenia did not find an increased risk of decompensation in cirrhosis patients [9,17]. Discrepancies in these findings may originate from dissimilar study populations, varied focus on outcomes, and differing criteria used to define sarcopenia. Our data strongly support that sarcopenia, defined as L3 SMI < 44.77 cm^2^/m^2^ and < 32.5 cm^2^/m^2^ in males and females, is an essential prognostic factor in patients with liver cirrhosis.

The reason why sarcopenia negatively affects variceal bleeding remains unclear. From a clinical point of view, portal hypertension degree is the most relevant predictor factor of EGVB. From a pathophysiological point of view, the relation of sarcopenia with portal hypertension might be bidirectional [13]. For instance, numerous portal hypertension-related factors, like the development of spontaneous portosystemic shunts, endotoxemia, and hyperammonemia, facilitate sarcopenia’s occurrence among cirrhosis patients [18]. Additionally, TIPS as an intervention for reducing portal pressure, has shown an improvement in muscle quantity and quality [19], proving that portal hypertension is an important contributor to sarcopenia development and its persistence. However, the role of sarcopenia in portal hypertension-related complications, including variceal bleeding is still debatable. Indeed, skeletal muscle is a secretory organ for cytokines and other polypeptides with autocrine, paracrine, and endocrine effects that are extensively involved in inflammatory processes [20]. Thus, sarcopenia may be related to persistent, low-grade inflammation, which increases with portal hypertension severity, circulatory dysfunction, and maximized complications like variceal bleeding [21,22]. From our results, we speculate that sarcopenia can promote the development of portal hypertension. Further studies are needed to confirm and expanding knowledge regarding the role of sarcopenia in portal hypertension.

Malnutrition in patients with liver cirrhosis is the result of a combination of factors, mainly related to reduced caloric intake, altered metabolism and malabsorption [23,24]. Previous studies have found that malnourished patients are at risk for more severe portal hypertension and variceal bleeding [25]. In addition, patients with cirrhosis complicated by malnutrition often develop sarcopenia, and sarcopenia responds to a state of protein-energy malnutrition in patients with cirrhosis; assessment of sarcopenia may enhance estimation of the risk of malnutrition in patients with cirrhosis, thereby identifying patients in need of some nutritional interventions before and after endoscopic therapy to improve outcomes. A randomized controlled trial demonstrated that supplements including branched-chain amino acids and nutrient-energy supplements were beneficial for cirrhotic patients undergoing endoscopic therapy [26]. Furthermore, dietary restrictions play a crucial role in endoscopic therapy, which worsens malnutrition and muscle loss in patients with cirrhosis. To prevent additional breakdown of proteins, it is important to avoid prolonged fasting periods. Our previous study’s findings indicate that early initiation of enteral nutrition is not only safe but also beneficial for patients’ recovery following endoscopic treatment compared to a 48-h postoperative fasting period [27]. More prospective studies are needed to explore whether improving the nutritional status of cirrhotic patients with sarcopenia may improve their prognosis.

In this study, the Hb level, NSBBs, and Child-Pugh scores independently predicted the risk factors for rebleeding after endoscopic therapy. Levy et al. found that a low Hb level might strongly predict infections [28]. It has been recognized that infection determines the rebleeding rate among cirrhosis patients because it elevates portal pressure *via* vasoactive substances [29]. Therefore, a low Hb level is a reasonable risk factor for rebleeding after endoscopic treatment. We also observed patients using NSBBs showed a decreased rebleeding rate when compared with patients not using them (*p* < 0.05). This might be due to the reductions in the portal blood flow and pressure by NSBBs. A recent meta-analysis showed higher rebleeding and death risks among patients receiving EVL alone compared to EVL + NSBBs [30]. The association between higher Child-Pugh scores and increased rebleeding risk after endoscopic treatment has been reported previously [31]. Similarly, we found that a higher Child-Pugh score significantly impacted rebleeding. We also found that compared with patients without sarcopenia, patients with cirrhosis and sarcopenia exhibited reduced liver function. Consequently, an improved liver function can improve the overall condition of patients and reduce the rebleeding risk.

Our results showed that the 2-year mortality rate decreased compared with other previously reported studies [31,32]. This might be because of several reasons: firstly, novel technologies and medications have been developed recently for efficiently decreasing portal hypertension while preventing mortality; secondly, it was the first endoscopic therapy for our patients; most patients in the cohort were Child-Pugh class A or B and only 2.9% were in Child-Pugh class C; thirdly, patients with a shorter follow-up duration and those who underwent liver transplantation during the follow-up period were categorized as alive; finally, we excluded hepatocellular carcinoma patients as it is related to poor survival in variceal bleeding patients. Therefore, we did not find a relationship between sarcopenia and a higher mortality rate.

Our study had a few limitations. This was a retrospective study that might have selection bias since we had enrolled patients who had CT images. Nonetheless, this study enhanced statistical reliability while reducing selection bias by PSM. Additionally, because of a short follow-up period, no mature survival data were obtained. Consequently, more studies with a longer follow-up period are warranted for verifying our results and assessing a long-time prognosis. Lastly, a majority of our patients had mild-to-moderate liver diseases, and these results do not apply to patients with Child–Pugh classes C.

## 5. Conclusions

In conclusion, this study is the first research work to suggest that sarcopenia is significantly and independently related to a high 2-year rebleeding rate in patients who received endoscopic therapy for preventing variceal rebleeding. As sarcopenia is an indicator of nutritional status, this study further emphasizes the importance of early screening for sarcopenia and necessary nutritional support in patients with cirrhosis, which can be used to improve muscle mass through appropriate dietary interventions and exercise, thereby improving clinical outcomes.

## Data Availability

The authors will supply the relevant data in response to reasonable requests.
